# Dendritic cell-derived VEGF-A plays a role in inflammatory angiogenesis of human secondary lymphoid organs and is driven by the coordinated activation of multiple transcription factors

**DOI:** 10.18632/oncotarget.9684

**Published:** 2016-05-31

**Authors:** Valentina Salvi, William Vermi, Veronica Gianello, Silvia Lonardi, Vincenzo Gagliostro, Antonella Naldini, Silvano Sozzani, Daniela Bosisio

**Affiliations:** ^1^ Department of Molecular and Translational Medicine, University of Brescia, Brescia, Italy; ^2^ Humanitas Clinical and Research Centre, Rozzano, Italy; ^3^ Department of Molecular and Developmental Medicine, University of Siena, Siena, Italy

**Keywords:** PAMPs, DAMPs, draining lymph node, CD1a+ interdigitating DCs, CD1c+ DCs, Pathology Section

## Abstract

Lymph node expansion during inflammation is essential to establish immune responses and relies on the development of blood and lymph vessels. Previous work in mice has shown that this process depends on the presence of VEGF-A produced by B cells, macrophages and stromal cells. In humans, however, the cell types and the mechanisms regulating the intranodal production of VEGF-A remain elusive. Here we show that CD11c^+^ cells represent the main VEGF-A-producing cell population in human reactive secondary lymphoid organs. In addition we find that three transcription factors, namely CREB, HIF-1α and STAT3, regulate the expression of VEGF-A in inflamed DCs. Both HIF-1α and STAT3 are activated by inflammatory agonists. Conversely, CREB phosphorylation represents the critical contribution of endogenous or exogenous PGE_2_. Taken together, these results propose a crucial role for DCs in lymph node inflammatory angiogenesis and identify novel potential cellular and molecular targets to limit inflammation in chronic diseases and tumors.

## INTRODUCTION

VEGF-A is a potent pro-angiogenic mediator typically released under hypoxic conditions, but is also a hallmark of inflammation [1–3]. The regulation of VEGF-A secretion by hypoxia has been thoroughly characterized (reviewed in [4, 5]). Far less is known about its regulation by pro-inflammatory stimuli, although transcription seems to play a predominant role [5]. The VEGF-A promoter spans more than 2 kb upstream of the transcription start site [6] and comprises many phylogenetically conserved binding sites for transcription factors such as HIF-1α, STAT3, CREB, nuclear receptors and Sp1 [5]. Among these, HIF-1α is recognized as the master regulator of VEGF-A transcription, although it is now clear that other transcription factors are required to support its key function [7].

During inflammation, draining lymph nodes rapidly enlarge and increase in cellularity to foster the encounter between antigens and immune cells. Lymph node swelling is supported by a dramatic increase in blood vasculature to provide oxygen, nutrient and immune cell delivery [8] and by robust lymphangiogenesis that further enhances the possibilities of DC-T lymphocyte interactions [9] [10]. The mechanisms regulating the growth of blood and lymphatic vasculature in lymph nodes are not completely understood, but several studies point to a pivotal role for VEGF-A [11] [12] [13]. The intranodal source of VEGF-A has been investigated in the mouse system and comprises follicular B cells [14, 15], CD11b^+^ macrophages [16, 17] and reticular stromal cells [18]. Also CD11c^+^ DCs were suggested to have an indirect role in the increase of intranodal VEGF-A [13]. In humans, the cellular source of VEGF-A in inflamed lymph nodes remains completely elusive [19].

DCs are antigen presenting cells located at the interface between innate and adaptive immunity. DCs express a vast repertoire of pattern recognition receptors and the binding of pathogen- and damage-associated molecular patterns (PAMPs and DAMPs) activates a complex pro-inflammatory program that contributes to the local inflammatory response [20]. After antigen uptake in peripheral tissues, DCs travel *via* afferent lymphatics to the draining lymph nodes where they activate antigen-specific T lymphocytes [21].

We have previously reported that human monocyte-derived DCs represent an important source of biologically active VEGF-A_165_ and VEGF-A_121_ [22]. This work investigates the ability of DCs to produce VEGF-A in human activated lymph nodes and characterizes the molecular mechanisms responsible for VEGF-A transcription under inflammatory conditions.

## RESULTS

### CD11c^+^ cells produce VEGF-A in human inflamed secondary lymphoid organs

**Figure 1:**
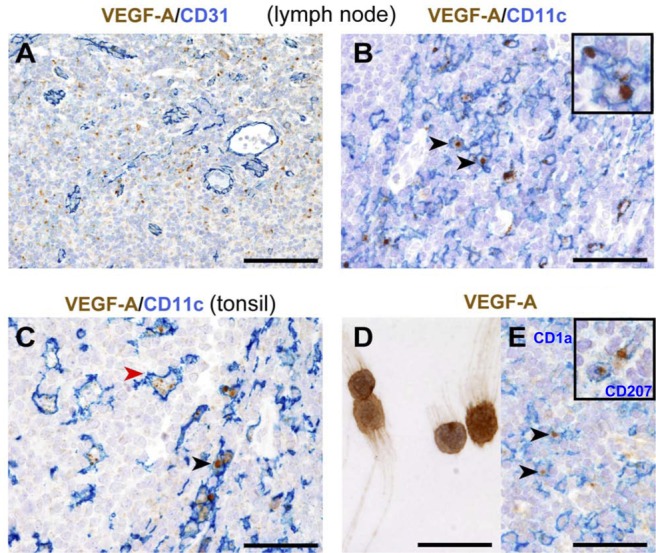
Distribution and phenotype of VEGF-A-producing cells in human reactive lymphoid tissues **A.** In reactive lymph nodes, VEGF-A^+^ cells surround CD31^+^ HEVs and **B.** co-express CD11c. **C.** In tonsils, strong cytoplasmic signal for VEGF-A is observed in CD11c^+^ cells in the interfollicular area (red arrow head) and CD11c+ germinal centre macrophages (black arrow head). **D.** Cytospin preparation of CD1c^+^ DCs sorted from tonsils shows VEGF-A-reactivity. **E.** In dermatopathic lymphadenitis, strong cytoplasmic VEGF-A is clearly detected in CD1a^+^ and CD207^+^ (inset) interdigitating DCs. Sections are from FFPE reactive lymph nodes (**A.**, **B.**, **E.)** and tonsil (C). Stainings are indicated by labels. Representative double positive cells in each panel are indicated by arrow heads and detailed at a high power view insets. Sections are counterstained with Meyer's haematoxylin. Original magnifications: 200X (**A.**, scale bar 100 μm), 400X (**B.**, **C.**, **E.** scale bar 50 μm), 600X (**D.** and insets, scale bar 33 μm).

Staining of reactive tonsils and lymph nodes, including tumor-draining lymph nodes, with an anti-VEGF-A antibody revealed a strong intracytoplasmic granular reactivity surrounding CD31^+^ high endothelial venules (HEVs) (Figure 1A). These VEGF-A^+^ cells represented a fraction of CD11c^+^ cells in both lymph nodes (Figure 1B) and tonsils (Figure 1C), whereas pDCs, B and T lymphocytes were negative (Supplemental Figure 1A, B and C). CD11c^+^ cells comprise both macrophages and myeloid DCs (mDCs), the latter being identified as CD1c^+^. Since an anti-CD1c antibody working on formalin-fixed tissue sections is currently not available, the expression of VEGF-A by mDCs was confirmed in CD1c^+^ mDCs freshly purified from human tonsils (Figure 1D). Of note, as reported in previous studies, CD1c^+^ cells represent the majority of HLADR^+^CD11c^+^ cells in reactive tonsils and lymph nodes (data not shown) [23, 24]. In addition to mDCs, VEGF-A reactivity was detected in CD1a^+^ and CD207^+^ interdigitating DCs (Figure 1E and inset, respectively and in some CD163^+^ macrophages (Supplemental Figure 1D). In reactive tonsils, a minor fraction of M-DC8/DD1^+^ slanDCs costained for VEGF-A (Supplemental Figure 1E).

Collectively, these data indicate that myeloid DC subsets, interdigitating DCs of Langerhans cell derivation and macrophages represent a major source of VEGF-A in human inflamed secondary lymphoid organs.

### The release of VEGF-A induced by pro-inflammatory stimuli depends on the presence of PGE_2_

**Figure 2:**
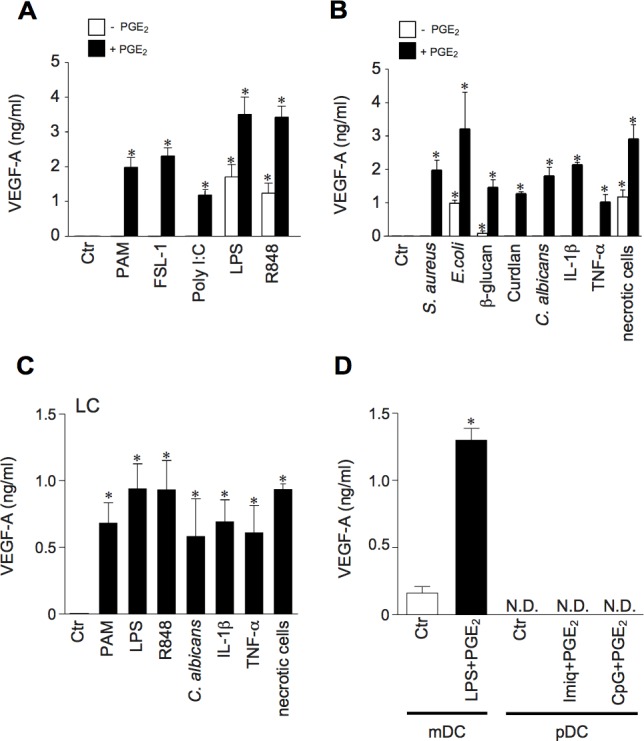
Human myeloid DCs produce VEGF-A in response to a variety of pro-inflammatory stimuli, provided PGE^2^ is present in the microenvironment **A.**, **B.** DCs were stimulated for 24 hours with TLR-ligands PAM_3_CSK_4_ (TLR1/2, 100 ng/ml), FSL-1 (TLR2/6, 100 ng/ml), Poly I:C (TLR3, 25 μg/ml), LPS (TLR4, 100 ng/ml) and R848 (TLR7 and TLR8, 5 μg/ml), Heat-killed *S.aureus* (specific for TLR2; 1:10 DC/bacteria ratio), E. coli (specific for TLR4; 1:10 DC/bacteria ratio), β-glucan (10 μg/ml), Curdlan (10 μg/ml), heat-killed *C.albicans* (specific for C-type lectins; 1:10 DC/fungi ratio), IL-1β (20 ng/ml), TNF-α (20 ng/ml), and necrotic cells (1:2 DC/necrotic cells ratio) in the presence or absence of PGE_2_ (10 μM). VEGF-A production was evaluated in cell-free supernatants by ELISA. Data are expressed as mean + SEM (*n* = 4); * *p* < 0.05 by one-way ANOVA with Dunnet's post hoc test. **C.** Langerhans cells (LCs) were stimulated for 24 hours with PAM_3_CSK_4_ (100 ng/ml), LPS (100 ng/ml), R848 (5 μg/ml), heat-killed *C. albicans (*1:10 DC/fungi ratio), IL-1β (20 ng/ml), TNF-α (20 ng/ml), and necrotic cells (1:2 DC/necrotic cells ratio) in the presence of 10 μM PGE_2_. VEGF-A production was evaluated by ELISA. Data are expressed as mean + SEM (*n* = 3). * *p* < 0.05 by one-way ANOVA with Dunnet's post hoc test **D.** Blood purified mDCs and pDCs were stimulated as indicated for 24 hours and VEGF-A levels were analyzed by ELISA. Data are expressed as mean + SEM (*n* = 3); * *p* < 0.05 by Student's *t* test; N.D. = Not Detectable.

The ability of DCs to produce VEGF-A in response to different pro-inflammatory mediators was further investigated *in vitro*. Monocyte-derived DCs were stimulated with Toll Like Receptor-(TLR) ligands (Figure 2A), or whole bacteria (*Staphilococcus aureus, Escherichia coli*), C-type lectin ligands (β-glucan, Curdlan and *Candida albicans*), pro-inflammatory cytokines (IL-1β and TNF-α) and necrotic cell-associated DAMPs (Figure 2B). Among these, only the ligands for TLR4 and TLR7-8, necrotic cell-associated DAMPs and, although at a low extent, β-glucan induced the release of VEGF-A. On the other hand, all stimuli became active in the presence of PGE_2_, the predominant eicosanoid produced in inflamed tissues and growing tumors [25]. Of note, PGE_2_ by itself did not induce VEGF-A (Figure 2A and 2B). According to the results obtained in tissue section, similar inductions were also obtained with *in vitro* differentiated Langerhans cells (Figure 2C) and primary mDCs (Figure 2D). Also, blood purified pDCs did not produce VEGF-A under basal conditions or when stimulated with TLR7- or TLR9-ligands either in the presence or absence of PGE_2_ (Figure 2D).

**Figure 3:**
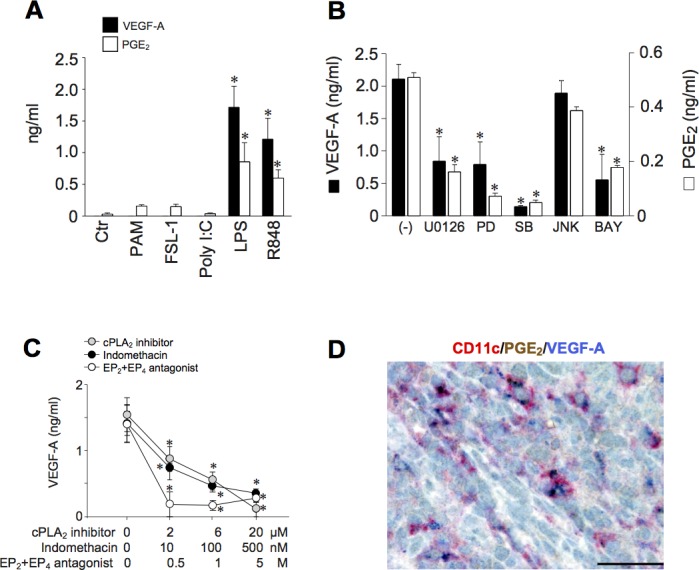
TLR-4-dependent expression of VEGF-A depends on endogenous PGE_2_ production **A.** DCs were stimulated with PAM_3_CSK_4_ (100 ng/ml), FSL-1 (100 ng/ml), Poly I:C (25 μg/ml), LPS (100 ng/ml) and R848 (5 μg/ml) for 24 hours. The production of VEGF-A and PGE_2_ was evaluated in cell-free supernatants by ELISA or EIA, respectively. Results are expressed as mean + SEM (*n* = 4); *p < 0.05 by one-way ANOVA with Dunnet's post hoc test. **B.** DCs were pre-treated for 1 hour with U0126 (1 μM), PD98059 (PD, 1 μM), SB203580 (SB, 1 μM), JNK Inhibitor II (JNK, 1 μM), BAY-11-7082 (BAY, 1 μM) and then stimulated with LPS (100 ng/ml) for 24 hours. VEGF-A and PGE_2_ production was evaluated by ELISA or EIA, respectively. Results are expressed as mean + SEM (*n* = 3); *p < 0.05 by one-way ANOVA with Dunnet's post hoc test. **C.** DCs were pre-treated for 1 hour with increasing concentrations of AACOF3 (cPLA_2_ inhibitor, grey circles), indomethacin (a non-selective COX-1/COX-2 inhibitor, black circles) or AH 6809+GW 627368X (EP_2_ and EP_4_ antagonist respectively, open circles) and then stimulated with LPS. After 24 hours, supernatants were collected and the production of VEGF-A evaluated by ELISA. Data are expressed as mean + SEM (*n* = 3); *p < 0.05 by one-way ANOVA with Dunnet's post hoc test. **D.** In reactive lymph nodes, a fraction of CD11c^+^ cells costains for VEGF-A and PGE_2_. Sections are counterstained with Meyer's haematoxylin. Original magnification: 600X (scale bar 33 μm).

Since LPS induces PGE_2_ synthesis [26], a role for endogenous PGE_2_ in inflammatory VEGF-A production was postulated. In accordance with this hypothesis, we found a clear correspondence between the capability to induce endogenous PGE_2_ and VEGF-A production (Figure 3A). Furthermore, the production of both mediators was ihibited by U0126, PD98059, SB203580 and BAY11-7082 (inhibitors of mitogen-activated protein kinase kinases, extracellular-signal-regulated kinase 1/2 (ERK1/2), MAPK p38 and NF-κB respectively), but not by the JNK inhibitor II (Figure 3B), suggesting the regulation by a common signalling pathways. Similar results were obtained with R848-stimulated DCs (data not shown). The role of endogenous PGE_2_ in VEGF-A secretion was further substantiated by two experimental approaches. First, by using specific inhibitors for two enzymes located upstream of PGE_2_ production, namely phospholipase A_2_ (PLA_2_) and COXs. Figure 3C shows that AACOF3 (a calcium-dependent PLA_2_ inhibitor), as well as indomethacin (a pan-COX-1/2 inhibitor), inhibited in a concentration-dependent manner VEGF-A production in LPS-stimulated DCs. As expected, both inhibitors also abolished the release of endogenous PGE_2_ (data not shown). We found that DCs express basal levels of Prostaglandin E receptor 2 and 4 (EP_2_ and EP_4_) that are not modulated by LPS stimulation (Supplemental Figure 2A). These receptors are functional and mediate DC response to PGE_2_, because Butaprost (an EP_2_ agonist) and Misoprostol (a promiscuous EP_2-4_ agonist) recapitulated PGE_2_ effects in LPS-stimulated DCs (Supplemental Figure 2B). Thus, as a second approach, cell activation by autocrine PGE_2_ was blocked by the use of specific inhibitors of EP_2_ and EP_4_. Figure 3C shows that AH 6809+GW 627368X (EP_2_ and EP_4_ antagonists, respectively) decreased VEGF-A production in a concentration-dependent manner.

Therefore, the ability of TLR-ligands to induce VEGF-A secretion directly correlates with the ability to activate PGE_2_ synthesis. This autocrine mechanism is supported by *in vivo* observations documenting the colocalization of anti-VEGF-A and anti-PGE_2_ immunoreactivity in CD11c^+^ cells (Figure 3D).

### Inflammatory VEGF-A transcription requires the coordinated activation of multiple transcription factors

**Figure 4:**
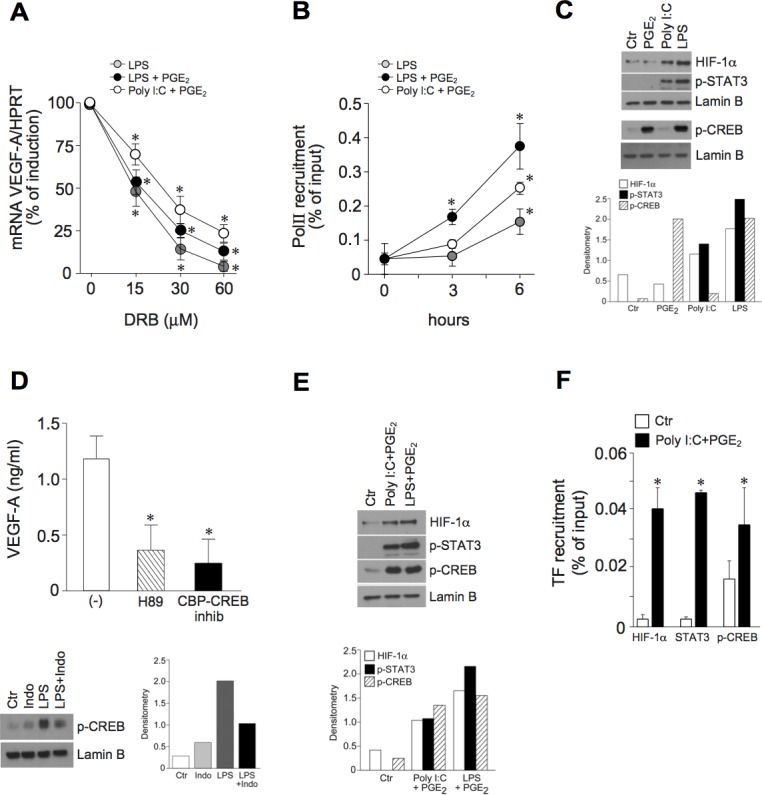
The pro-inflammatory VEGF-A transcription correlates with the activation of HIF-1α, STAT3 and CREB **A.** DCs were pre-treated with increasing concentrations of RNApolII inhibitor DRB for 30 minutes and then stimulated with LPS (6 hours), LPS+PGE_2_ or Poly I:C+PGE_2_ (3 hours). VEGF-A mRNA levels were evaluated by real-time PCR. Data are presented as mean + SEM (*n* = 3) of percentage of induction (100% indicates peak mRNA levels for each individual stimulus); *p < 0.05 by one-way ANOVA with Dunnet's post hoc test. **B.** DCs were stimulated with LPS, LPS+PGE_2_ or Poly I:C+PGE_2_ for the indicated time-points. Sonicated nuclear fractions were subjected to ChIP with anti-RNApolII (PolII) antibody and analyzed by RT-PCR. Data are shown as the immunoprecipitated percentage of input DNA and are expressed as mean + SEM (*n* = 3); * *p* < 0.05 compared with respective controls by one-way ANOVA with Dunnet's post hoc test. **C.**, **E.** DCs were stimulated with 10 μM PGE_2_, 25 μg/ml Poly I:C and 100 ng/ml LPS for 6 hours (**C.**, upper panels) or 30 minutes (**C.**, lower panels) or Poly I:C+PGE_2_ or LPS+PGE_2_ for 3 hours (**E)** and blotted against HIF-1α, phospho-STAT3 or phospho-CREB. Lamin B represents the loading control for nuclear proteins. One representative fluorogram out of three and its densitometric analysis are shown. The time points shown in the figure represent the peak intensity of kinetic stimulations of 30 minutes, 1, 3, 6 and 8 hours. One representative experiment out of 3 is shown. **D.** (upper panel) DCs were pre-treated for 1 hour with 5 μM H89 (PKA inhibitor, striped bar) or 3 μM CBP-CREB interaction inhibitor (black bar) and stimulated with LPS for 24 hours. VEGF-A production was evaluated by ELISA. Results are expressed as mean + SEM (*n* = 3); *p < 0.05 by one-way ANOVA with Dunnet's post hoc test. (**D.**, lower panel) DCs were pre-treated for 30 minutes with 100 nM indomethacin and then stimulated with LPS. Nuclear extracts were blotted against phospho-CREB. Lamin B represents the loading control for nuclear proteins. One representative fluorogram out of three and its densitometric analysis are shown. **F.** Nuclear fractions of DCs stimulated with Poly I:C+PGE_2_ for 3 hours were subjected to chromatin immunoprecipitation with antibodies directed against HIF-1α, phospho-CREB and STAT3. Data are shown as percentage of immunoprecipitated DNA and are expressed as mean + SEM (*n* = 3); * *p* < 0.05 by Student's *t* test.

These results prompted us to investigate the molecular mechanisms involved in the combined activation of DCs by TLR ligands and PGE_2_. Studies were focused on the combination Poly I:C+PGE_2_ as the paradigm of a positive interaction of two agonists that are by themself inactive in VEGF-A production, while LPS was selected as the prototypic active agonist. The induction of VEGF-A mRNA was largely dependent on the activation of gene transcription, as shown by the use of the RNA polymerase II (RNApolII) inhibitor 5,6-Dichloro-1-β-D-ribofuranosylbenzimidazole (DRB), which dose-dependently decreased the peak levels of VEGF-A mRNA (Figure 4A) and by RNApolII recruitment to the VEGF-A promoter by chromatin immunoprecipitation (ChIP) (Figure 4B).

Since PGE_2_ by itself does not induce VEGF-A production, it is likely to contribute to the activation of a transcription factor that is crucial, but not sufficient *per se*, for the inflammatory production of VEGF-A. HIF-1α, STAT3 and CREB were selected for further analysis among the transcription factors able to bind the VEGF-A promoter [5] and known to be activated by TLRs and PGE_2_. Poly I:C and LPS, but not PGE_2_, induced the nuclear accumulation of HIF-1α as well as the phosphorylation and nuclear translocation of STAT3 (Figure 4C, upper panels). By contrast, PGE_2_ induced the rapid phosphorylation of CREB (Figure 4C, lower panels). Of note LPS, the only tested agonist with the ability to induce VEGF-A directly, also induced the phosphorylation of CREB, suggesting that only the concomitant presence of these three transcription factors can promote the activation of VEGF-A transcription. According to this hypothesis, both a specific CREB inhibitor and H89, an inhibitor of the CREB-phosphorylating enzyme PKA, strongly reduced the secretion of VEGF-A in LPS-stimulated DCs (Figure 4D, upper panel). Also, indomethacin reduced the LPS-dependent phosphorylation of CREB, thus demonstrating that endogenous PGE_2_ is responsible for CREB phosphorylation (Figure 4D, lower panel). Of note, the combined stimulation of DCs with Poly I:C+PGE_2_ induced the concomitant nuclear translocation of all transcription factors (Figure 4E) and their recruitment to the VEGF-A promoter (Figure 4F). Similar results were obtained in LPS+PGE_2_-stimulated DCs (Figure 4E and Supplemental Figure 3A).

**Figure 5:**
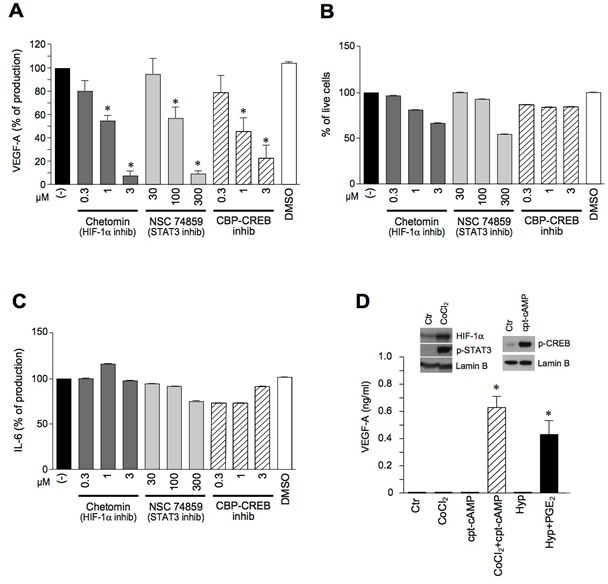
The pro-inflammatory secretion of VEGF-A requires the concomitant activation of HIF-1α, STAT3 and CREB **A.**-**C.** DCs were pre-treated for 1 hour with Chetomin, NSC 74859 or CBP-CREB inhibitor and stimulated with Poly I:C+PGE_2_ for 24 hours. The release of VEGF-A (**A**) or IL-6 (**C**) was evaluated by ELISA. Cell viability was evaluated by Propidium Iodide staining (**B**). **D.** DCs were stimulated with 400 μM CoCl_2_, 25 μM cpt-cAMP alone or in combination and in hypoxic conditions (Hyp) in the presence or absence of 10 μM PGE_2_ for 24 hours. The production of VEGF-A was evaluated by ELISA in cell free supernatants. Results are expressed as mean + SEM (*n* = 3). * *p* < 0.05 compared to respective controls by Student's *t* test or one-way ANOVA with Dunnet's post hoc test as appropriate. (**D.**, left inset) DCs were stimulated with 400 μM CoCl_2_ for 4 hours or (**D.**, right inset) with 25 μM cpt-cAMP for 30 minutes and blotted against HIF-1α, phospho-STAT3 or phospho-CREB. Lamin B represents the loading control for nuclear proteins.

The requirement of these three transcription factors for VEGF-A production was further confirmed by the use of specific inhibitors. Figure 5A shows that Chetomin (an inhibitor of the interaction of HIF-1α with transcriptional co-activators p300 and CBP), NSC 74859 (a STAT3 inhibitor) and CBP-CREB (a CREB inhibitor) dose-dependently reduced the secretion of VEGF-A after Poly I:C+PGE_2_ stimulation. Although with the highest concentrations of the first two inhibitors a minor reduction in cell viability was observed at the end of 24 hour-stimulation (Figure 5B), the ability of the cells to release IL-6, an unrelated cytokine, was not affected (Figure 5C). These results strongly suggest that the inhibition of VEGF-A production was not due to the toxic effects of the inhibitors. Similar results were obtained in LPS+PGE_2_-stimulated DCs (Supplemental Figure 3B-3D). Finally, the involvement of multiple transcription factors in VEGF-A induction was further strengthened by the use of chemical activators. Figure 5D shows that, in human DCs, CoCl_2_ (a drug activating HIF-1α and STAT3, left inset [27]) or hypoxia, were by themselves inactive and induced VEGF-A only when combined with the CREB-phosphorylating agonist (8-(4-Chlorophenylthio)-cAMP (cpt-cAMP), or PGE_2_.

These results indicate that the transcriptional activation of VEGF-A in inflamed DCs requires the concomitant activation of multiple transcription factors, namely CREB, HIF-1α and STAT3, and that CREB phosphorylation represents the crucial contribution of PGE_2_ to TLR-dependent VEGF-A secretion.

## DISCUSSION

Activated DCs produce large amounts of chemokines, cytokines and oxygen radicals and represent an important component of the inflammatory reaction [21]. In adults, angiogenesis occurs mostly under pathological conditions, including inflammation. Newly formed blood vessels provide nutrients to growing tissues and allow accumulation of immune cells, both in peripheral tissues and in secondary lymphoid organs [1]. In this study we have identified the nature of VEGF-A producing cells in sections of human reactive lymph nodes and tonsils. Specifically, double-immunohistochemistry shows that CD1c^+^ mDCs and interdigitating DCs represent a major source of VEGF-A in inflamed secondary lymphoid organs. Interdigitating DCs originate from Langerhans cells migrated *via* lymphatics. On the other hand, CD1c^+^ mDCs, located in the extranodular compartments surrounding HEVs, likely migrated from the blood. Because of their different nodal location, each of these cell types may play a specific role in the neo-angiogenesis process of lymphatics sinuses and HEVs, respectively.

**Figure 6:**
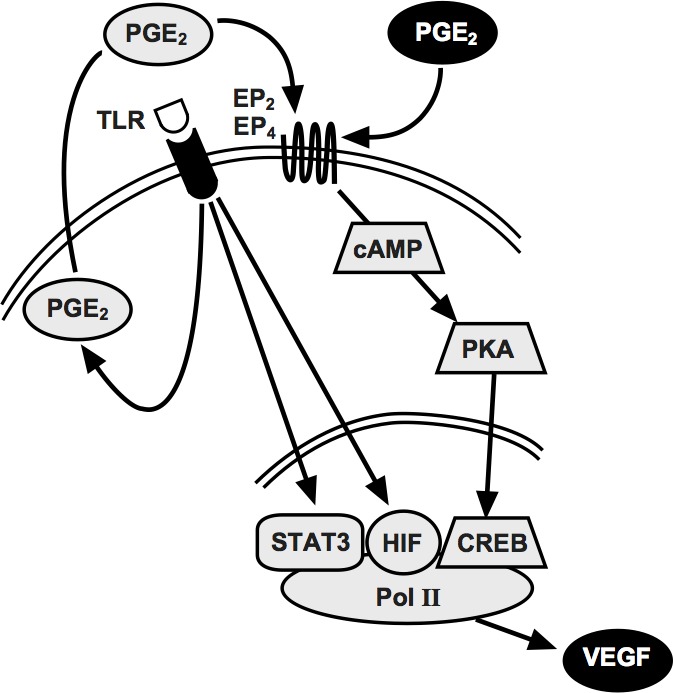
Proposed model for inflammatory VEGF-A production The triggering of TLR4 (or TLR7-8) induces the release of endogenous PGE_2_ (grey circle) which acts autocrinally on EP_2_ and/or EP_4_ receptors inducing robust CREB phosphorylation. This, together with TLR-activated HIF-1α and STAT3, finally induces VEGF-A transcription. For pattern recognition receptors that don't release PGE_2_, exogenous PGE_2_ (black circle) provides CREB phosphorylation.

Our results from *in vitro* studies show that DCs can readily produce VEGF-A in response to a large variety of PAMPs and DAMPs if PGE_2_ is present in the local microenvironment, demonstrating a non-redundant role for PGE_2_ in VEGF-A production by DCs. This PGE_2_ may either be endogenously produced by DCs, or provided together with the stimuli (outlined in Figure 6). Despite PGE_2_ concentrations shown in Figure 2A and 2B were higher than those released by DCs (Figure 3A), similar results were obtained with PGE_2_ concentrations resembling those autocrinally released (0.01 μM, not shown). The presence of CD11c/VEGF-A/PGE_2_ positive cells in tissue sections supports the existence of an autocrine mechanism *in vivo*. In addition PGE_2_, which is abundantly produced by many cell types in inflamed tissues and lymph nodes [25, 11, 12], may also act paracrinally to support VEGF-A production in the presence of PAMPs or DAMPs unable to induce PGE_2_ in DCs. PGE_2_ regulates several DC functions including migration, edema formation and T cell polarization [25] [28] [29] [30]. This report further strengthen the importance of PGE_2_ in DC biology showing the crucial role of this prostenoid for VEGF-A production. Furthermore, this study provides an additional mechanism by which COX-2 may promote inflammatory and/or tumor angiogenesis [31].

From a mechanistic point of view, our data propose CREB phosphorylation as the crucial contribution of PGE_2_ to VEGF-A secretion, while TLRs are responsible for the activation of HIF-1α and STAT3. The mechanisms of HIF-1α and STAT3 activation by TLRs remain unexplained. It was previously described in cancer cell lines that pro-inflammatory cytokines and TLR3-4 ligands induce HIF-1α accumulation under normoxic conditions [32–36], an effect often correlated with COX-2 activation. However, in DCs, HIF-1α accumulation does not depend on COX-2 activation since it is detected also after the stimulation with Poly I:C. In addition, we found that COX inhibition could not prevent HIF-1α accumulation in LPS-stimulated DCs (V.S., unpublished results). Based on these observations, we hypothesize that HIF-1α accumulation may depend on a direct effect of TLR signalling, possibly mediated by ROS that were described to inhibit PHD enzymes and stabilize HIF-1α protein [37]. The mechanisms of STAT3 activation are even more puzzling. A good candidate would be an autocrine loop involving IL-6, which is induced by several TLRs and is a well known VEGF-A inducer [5]. However, an IL-6R-blocking antibody did not inhibit the release of VEGF-A (V.S., unpublished results) in our system. Also, the production of VEGF-A was not affected by antibodies blocking the receptors for IL-10 and IFN-I (V.S., unpublished results), thus suggesting the involvement of a different mediator which remains elusive. Further work is granted to better characterize the mechanisms responsible for HIF-1α and STAT3 activation in TLR-stimulated DCs.

The conclusion that, in DCs, three transcription factors (namely HIF-1α, STAT3 and CREB) are required to induce VEGF-A transcription (outlined in Figure 6) is supported by experiments performed with specific inhibitors and by the observation that agonists which fail to induce the activation of these transcription factors also fail to induce VEGF-A transcription, unless combined with exogenous PGE_2_. This is the case for TLR3 activation, which accumulates HIF-1α and phospho-STAT3 but fails to induce CREB phosphorylation. Similarly, CoCl_2_, which accumulates HIF-1α and phospho-STAT3, induces VEGF-A only when combined with CREB-activating drug cpt-cAMP. These latter findings suggest that the presence of three transcription factors is crucial not only in pro-inflammatory, but also in hypoxic conditions. In the past, seminal work postulated the need for a multiprotein complex, including HIF-1α and an “adjacent transcription factor” to regulate the transcription of hypoxic genes [38, 39]. In line with this, it was recently proposed that STAT3 may cooperatively activate HIF-1α-target genes by increasing the recruitment of the coactivators CBP and p300 [7, 27, 40]. Our results further support these findings showing the need for three transcription factors for the inflammatory production of VEGF-A.

An unresolved question is whether HIF-1α, STAT3 and CREB activate VEGF-A transcription dependently or independently from each other, i.e. if they form a protein complex (or enhanceosome) in human DCs. Even in the absence of direct evidence from co-occupancy experiments, several data strongly support this hypothesis. First, the three transcription factors can be detected on the same promoter region, encompassing functional HIF and STAT3 sites less than 150 bp apart one from the other. This region is devoid of a characterized CRE site, but a nearby (less than 200 bp) functional AP-1 site [5] may bind CREB because of core consensus sequence similarities [41]. Alternatively, the binding of CREB to the VEGF-A promoter may be indirect, i.e. mediated by coactivators as previously proposed [42]. Finally, we show that the inhibition of each single transcription factor completely flattens the secretion of VEGF-A. This suggests the existence of a protein complex encompassing the three transcription factors, which cannot be assembled in the lack of one member, thus failing to recruit co-activators and to activate transcription.

In conclusion, this study shows that, in inflammatory conditions, human DCs can produce conspicuous amounts of VEGF-A, *in vitro* and *in vivo*. Our data also show that in inflamed lymph nodes DCs represent one of the few cell types contributing VEGF-A production. This finding makes DCs a new potential therapeutic target to limit lymph node vascular expansion in chronic- and tumor-associated inflammation. Furthermore, the formal definition of the enhanceosome composition might allow to specifically target DC-derived VEGF-A preserving the physiological angiogenesis as well as the global activation of transcription factors mastering key physiological processes. Altogether, this study further expands our knowledge on the plasticity of DCs and on their complex role in the regulation of innate immune responses.

## MATERIALS AND METHODS

### Immunohistochemistry

Formalin-fixed paraffin-embedded human tissues were retrieved from the archive of the Department of Pathology (Spedali Civili di Brescia, Brescia, Italy). Anti-VEGF-A polyclonal antibody (1:500, Thermo Scientific, CA) was applied to 4-μm thick tissue sections, after appropriate antigen retrieval (microwave oven 3×5 minutes 750W in EDTA buffer, pH 8.0); the reaction was revealed using Novolink Polymer (Leica Microsystems, UK) followed by DAB. Anti-PGE_2_ (polyclonal Rabbit, 1:700 over night, Biorbyt) was revealed using DakoEnvision+System-HRP Labelled Polymer Anti-Rabbit and DAB after antigen retrieval (thermostatic bath, TRIS-EDTA buffer, pH 9.0). Isotype control stains for both VEGF-A and PGE_2_ were performed and gave no reactivity. Characterization of VEGF-A positive cells was performed by double immunohistochemistry using antibodies to the following antigens: CD31 (mouse IgG1, clone 1A10, 1:50, Leica Microsystems), CD1a (mouse, clone 010, 1:50, Dako, Denmark), CD3 (rabbit, clone SP7, 1:100, Thermo Scientific), CD11c (mouse, clone 5D11, 1:50, Leica Microsystems), CD20 (mouse, clone L26, 1:250, Dako), CD163 (mouse, clone 10D6, 1:50, Thermo Scientific), CD207/Langerin (mouse, clone 12D6, 1:200, Vector Laboratories, CA) and CD303/BDCA2 (mouse, clone 124B3.13, 1:75, Dendritics, France) and M-DC8 (mouse, clone DD1, 1:70, kindly provided by K. Schäkel) and visualized using Mach 4 MR-AP (Biocare Medical, CA), followed by Ferangi Blue (Biocare Medical) as chromogen. Immunostained sections were photographed using the DP-70 Olympus digital camera mounted on the Olympus BX60 microscope.

### Cell preparation and culture

Buffy coats were obtained through the courtesy of the Centro Trasfusionale, Spedali Civili, Brescia. Monocytes were purified from peripheral blood mononuclear cells (PBMC) by immunomagnetic separation using anti CD14-conjugated magnetic microbeads (Miltenyi Biotec, Bergisch Gladbach, Germany). Monocyte-derived DCs were differentiated as previously described [43]. Briefly, monocytes cultured for 6 days in tissue culture plates in RPMI 1640 (Gibco, Invitrogen, Carlsbad, CA, USA) supplemented with 10% heat-inactivated fetal calf serum (FCS, Lonza Group, Switzerland), 2 mM L-glutamine, antibiotics (Gibco) (completed RPMI medium), 50 ng/ml GM-CSF and 20 ng/ml IL-4 (ProSpec Technogene, Israel). To generate Langerhans cells, monocytes were cultured for 6 days in completed RPMI medium in the presence of 50 ng/ml GM-CSF, 10 ng/ml IL-4 and 10 ng/ml TGF-β (Peprotech, London, U.K). Langerhans cell differentiation was controlled by monitoring Langerin and E-Cadherin expression. Myeloid DCs (mDCs) and plasmacytoid DCs (pDCs) were isolated using the corresponding Cell Isolation Kit (Miltenyi Biotec). pDCs were cultured in completed RPMI medium and 20 ng/ml IL-3 (ProSpec). Necrotic cells were obtained *via* four cycles of freezing and thawing. Hypoxia (1% O_2_, 37°C) was performed in the InVivo_2_ 400 hypoxic workstation (Ruskinn-Biotrace, UK).

### Cell stimulation

DCs (2×10^6^ cells/ml) were stimulated with the following agonists: 100 ng/ml PAM_3_CSK_4_, ligand for TLR1/2; 100 ng/ml FSL-1, ligand for TLR2/6; 25 μg/ml Poly I:C, ligand for TLR3; 100 ng/ml flagellin, ligand for TLR5 (*Bacillus subtilis*); 5 μg/ml imiquimod, ligand for TLR7; 5 μg/ml R848, ligand for TLR7 and TLR8 (from now on: TLR7-8); 6 μg/ml CpG ODN 2216, ligand for TLR9 (all from Invivogen, San Diego, California, USA); 100 ng/ml LPS, ligand for TLR4 (*Escherichia coli* 055:B5; Sigma-Aldrich, St. Louis, MO); 10 μg/ml β-glucan from baker's yeast (*Saccharomyces cervisiae*) or Curdlan (from *Alcaligenes faecalis*), both ligands for dectin-1 (both from Sigma-Aldrich); 20 ng/ml IL-1β and TNF-α (Peprotech). Heat-killed *Staphylococcus aureus, Escherichia coli* and *Candida albicans* was purchased from Invivogen. Where indicated, 10 μM PGE_2_, 25 μM cpt-cAMP, 400 μM Cobalt Chloride (CoCl_2_) were added (all from Sigma-Aldrich). DRB (an inhibitor of RNApolII), Chetomin (HIF-1α inhibitor), NSC 74859 (STAT3 inhibitor) were from Sigma-Aldrich; indomethacin (a non selective COX-1 and COX-2 inhibitor), U0126 (a MEK1/2 inhibitor), PD98059 (an ERK1/2 inhibitor), SB203580 (a p38 MAPK inhibitor), JNK Inhibitor II (a JNK inhibitor), BAY11-7082 (a NF-κB inhibitor), CBP-CREB interaction inhibitor were from Calbiochem (San Diego, CA); H89 (a PKA inhibitor), AH 6809 (an EP_2_ antagonist), GW 627368X (an EP_4_ antagonist), Butaprost (EP_2_ agonist), Misoprostol (EP_4_,EP_3_ > EP_2_ agonist) and Sulprostone (EP_1_/EP_3_ agonist) were from Cayman Chemical (Michigan, USA). AACOF3 (Arachidonyltrifluormethyl ketone, a cPLA_2_ inhibitor) was purchased from Biomol (Enzo Life Sciences, Farmingdale).

### Chromatin immunoprecipitation

After stimulation, cells were fixed by adding directly to the medium 36% formaldehyde (Sigma-Aldrich) to a final concentration of 1% for 7 minutes. The cross-linking reaction was stopped by adding Tris to a final concentration of 125 mM. After 10 minutes, ice-cold PBS was added and plates were transferred on ice, washed extensively with PBS, and scraped. After centrifugation, cells were lysed for 5 minutes in L1 buffer (50 mM Tris pH 8.0, 2 mM EDTA, 0.1% Nonidet P-40 and 10% glycerol) supplemented with inhibitors (1 mM Na3VO4, 2 mM DTT, 1 mM NaF, 1 mM PMSF and Protease Inhibitors Cocktail, all reagents were purchased from Sigma-Aldrich). Nuclei were pelleted at 3000 rpm in a cold microfuge and resuspended in L2 buffer (50 mM Tris pH 8.0, 1% SDS and 5 mM EDTA) plus inhibitors. Chromatin was sheared by sonication (3 × 20 s at 50% of the maximum potency), centrifuged to pellet debris, and diluted 10 times in dilution buffer (50 mM Tris pH 8.0, 0.5% Nonidet P-40, 200 mM NaCl and 5 mM EDTA). Extracts were precleared for 3 hours with a 50% suspension of salmon sperm-saturated protein A (ss protein A). Immunoprecipitations were carried out overnight at 4°C using 3 μg of the following antibodies raised against RNApolII (N-20 sc-899, Santa Cruz Biotechnology, CA, USA), HIF-1α (NB 10134, Novus Biologicals, CO, USA), STAT3 (C-20 sc-482, Santa Cruz Biotechnology) [44], pCREB^Ser133^ (Cat. 17-10131, Millipore; USA). Immune complexes were collected with protein A and washed three times (10 minutes each) with high salt buffer (washing buffer: 20 mM Tris pH 8.0, 0.1% SDS, 1% Nonidet P-40, 2 mM EDTA and 500 mM NaCl supplemented with 1mM PMSF) and three times with low salt buffer (1x Tris/EDTA [TE]). Immune complexes were extracted in 1x TE containing 2% SDS, and protein-DNA cross-links were reverted by heating at 65°C overnight. DNA was then extracted with the QIAquick PCR purification kit (Qiagen, Crawley, UK) and 1/20 of the immunoprecipitated DNA was used in each quantitative PCR reaction. Sequences of promoter-specific primers used were the following: VEGF-A −884 to detect transcription factor binding (forward: 5′-TGATGTCTGCAGGCCAGAT-3′, reverse: 5′-CCACAGTGTGTCCCTCTGAC-3′) and VEGF-A −291 to detect RNApolII recruitment (forward: 5′-GTCCGCACGTAACCTCACTT-3′, reverse: 5′-CAGCCTGAAAATTACCCATCC-3′). In all experiments, 10% of the input chromatin was removed prior to addition of the antibodies and used to normalize the amount of immunoprecipitated DNA (specified in the text as “input” DNA).

### ELISA

DCs were incubated for 24 hours with the indicated treatments. Cell-free supernatants were harvested and VEGF-A and IL-6 production was measured by ELISA assay (R&D Systems, Minneapolis, MN, USA). PGE^2^ production was assessed by EIA kit (Cayman Chemical).

### SDS-PAGE and western blot

Following the designated treatments, DCs were washed twice with PBS and lysed in L1 buffer with inhibitors to separate cytoplasmic proteins. Nuclear pellets were washed twice with L1 buffer with inhibitors and then lysed in Nonidet P-40 Lysis buffer (50 mM Tris-HCl, pH 8.0; 250 mM NaCl; 1 mM EDTA; 0.1% Nonidet P-40 and 10% glycerol) with inhibitors. Equal amounts of extracts were analyzed through 8-12% SDS-PAGE followed by Western blotting with antibodies against phospho-CREB (Cat. 9198, Cell Signalling), phospho-STAT3 (Cat. 9131, Cell Signalling), HIF-1α (Cat. 610959, BD Bioscience), and Lamin B (C-20 sc-6216, Santa Cruz Biotechnology). Protein bands were detected with SuperSignal West Pico Chemiluminescent Substrate (Pierce, Rockford, USA). Densitometric analysis was performed using ImageJ (version 1.48) software package from National Institutes of Health. Immunoblots were scanned as JPEG images and the areas under the curves were measured for each band and quantified. Data were normalized based on Lamin B content.

### Real-time PCR

RNA was extracted using TRIzol reagent (Invitrogen) according to the manufacturer's instructions. After RNA purification, samples were treated with DNAse to remove contaminating genomic DNA (DNaseI Amplification grade, Invitrogen). Reverse transcription was performed using random hexamers and MMLV RT (Invitrogen). Gene-specific primers used were as follows: hVEGF-A (forward: 5′-AGTGTGTGCCCACTGAGGA-3′, reverse: 5′-GGTGAGGTTTGATCCGCATA-3′), hHPRT (forward: 5′-CCAGTAACAGGGGACATAAA-3′, reverse: 5′-CACAATCAAGACATTCTTTCCAGT-3′). The SsoAdvanced Universal SYBR Green Supermix (Bio-Rad Laboratories Inc., Hercules, CA, USA) for quantitative real-time PCR was used according to the manufacturer's instructions. Reactions were run in triplicate on a StepOne Plus Real-Time PCR System (Applied Biosystems) and the generated products analyzed by the StepOne Plus Software (Version 2.3, Applied Biosystems). Gene expression was normalized based on HPRT mRNA content.

### FACS analysis

DCs were treated with 100 ng/ml LPS for 24 hours. At the end of the incubation, cells were permeabilized using the Cytofix/Cytoperm kit (BD Bioscience, San Diego, CA) and stained with antibodies against EP_2_ and EP_4_ receptors (Cayman Chemical) or with the isotype control for 30 minutes, washed with PBS (Gibco) and incubated with anti-rabbit Alexa-488 (Invitrogen). Samples were read on a PAS (Partec GmbH, Muenster, Germany) and analysed with FlowJo (Tree Star Inc, Ashland, USA). To assess cell viability, DCs were stained with Propidium Iodide (Invitrogen).

### Statistical analysis

Statistical significance among the experimental groups was determined using paired Student's *t* test or one-way ANOVA with Dunnet's post hoc test as appropriated (GraphPad Prism version 4.00 for Windows, GraphPad Software).

## SUPPLEMENTARY MATERIALS FIGURES


