# Supplementary material for: Dendritic cell-derived VEGF-A plays a role in inflammatory angiogenesis of human secondary lymphoid organs and is driven by the coordinated activation of multiple transcription factors
Source: Oncotarget. 2016 May 31;7(26):39256–69. doi: 10.18632/oncotarget.9684 (PMC5129930; doi:10.18632/oncotarget.9684)
Supplement: Supplementary file 1 [file oncotarget-07-39256-s001.pdf]

Dendritic cell-derived VEGF-A plays a role in inflammatory angiogenesis of human secondary lymphoid organs and is driven by the coordinated activation of multiple transcription factors

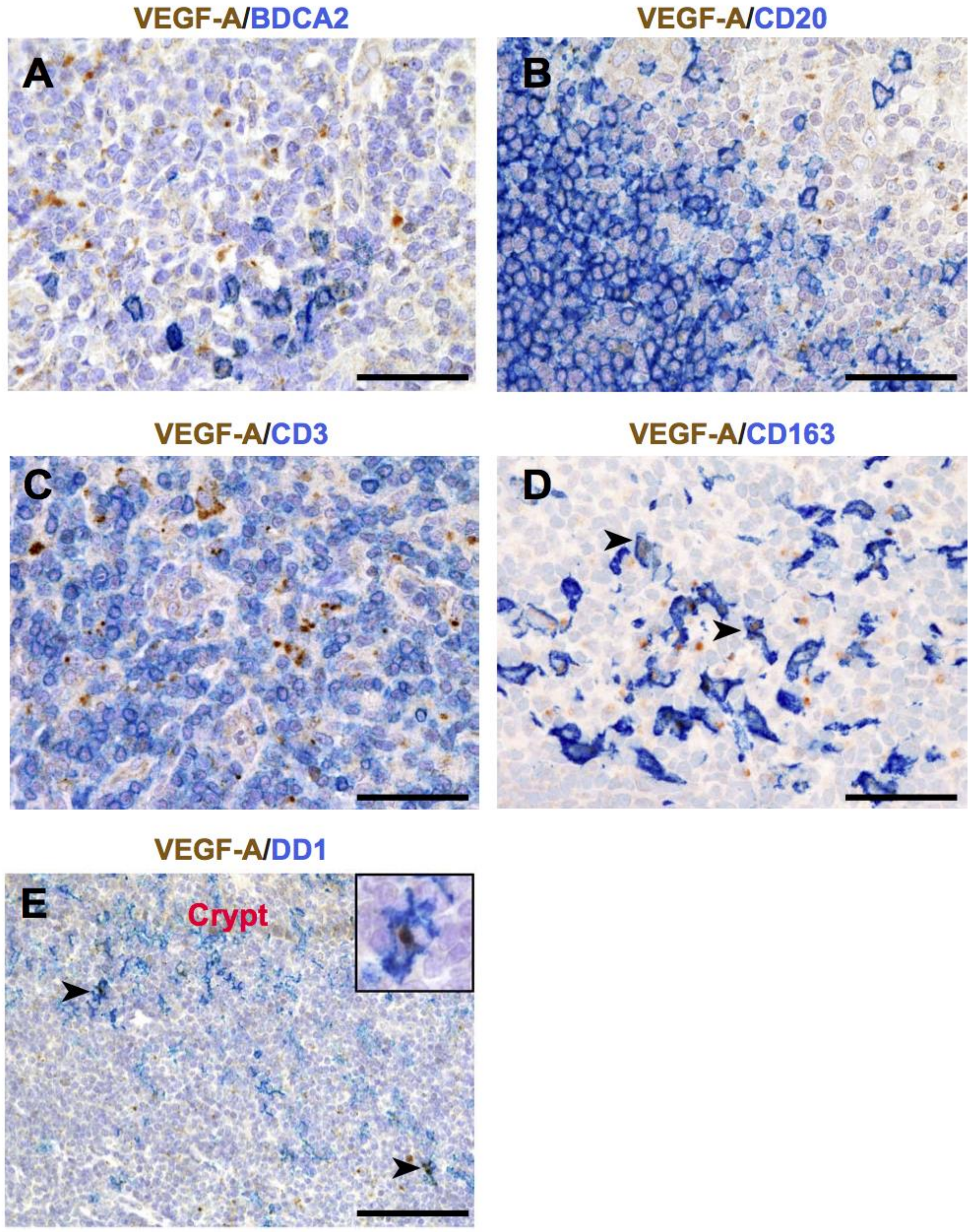

Supplemental Figure 1. Distribution and phenotype of VEGF-A producing cells in human reactive lympoid tissues.

(A) BDCA2+ plasmacytoid dendritic cells are negative for VEGF-A in dermatopathic lymphadenitis. **(B, C)** Nodal CD20+ B lymphocytes and CD3+ T cells are substantially negative. **(D)** In lymph node, VEGF-A stains also CD163+ macrophages (arrow heads). **(E)** In tonsils, DD1+ slanDCs stain for VEGF-A. Crypt= tonsil crypt epithelium. Section from fixed human lymph nodes (A-D) and tonsil (E) are stained as indicated by labels. Sections are counterstained with Meyer's haematoxylin. Original magnifications: 200X (E, scale bar 100 µm); 400X (A-D, scale bar 50 µm).

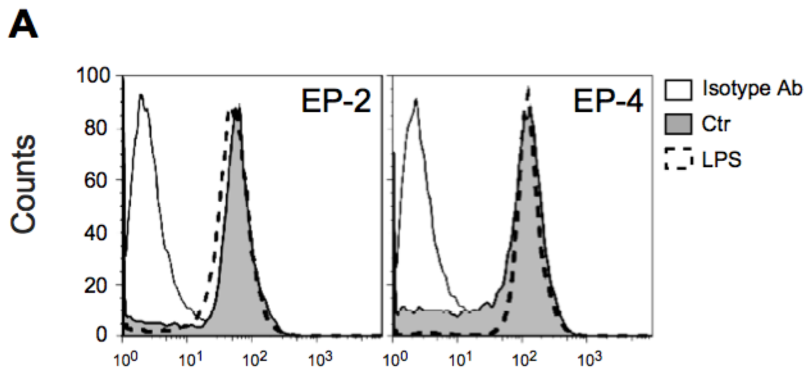

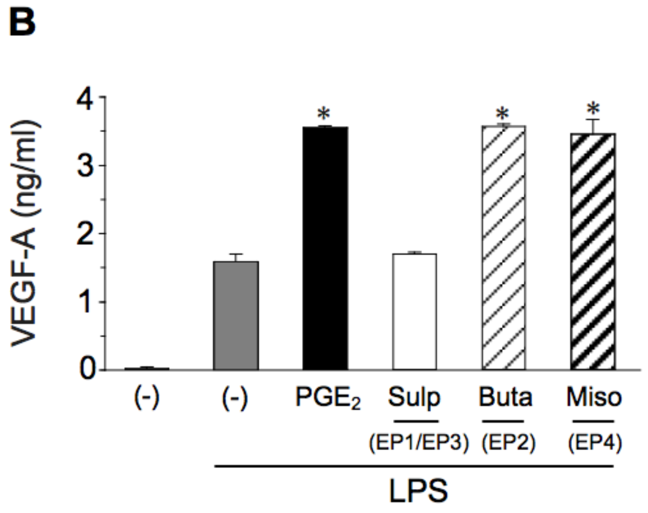

Supplemental Figure 2. EP_2 and EP_4 receptors mediate DC response to PGE_2.

(A) The levels of EP_2 and EP_4 receptors were examined by flow cytometry in unstimulated (grey area) or LPS-stimulated DCs (dashed line) as compared to isotype control (white area).

One representative experiment out of 3 is shown. **(B)** Butaprost (EP_2 agonist, 10 μM) and Misoprostol (EP_4,EP_3>EP_2 agonist, 10 μM) recapitulated the effect of PGE_2, while Sulprostone (EP_1/EP_3 agonist, 10 μM) did not. Results are expressed as mean $\pm$ SEM (n=3); *P< 0.05 by one-way ANOVA with Dunnet's post hoc test.

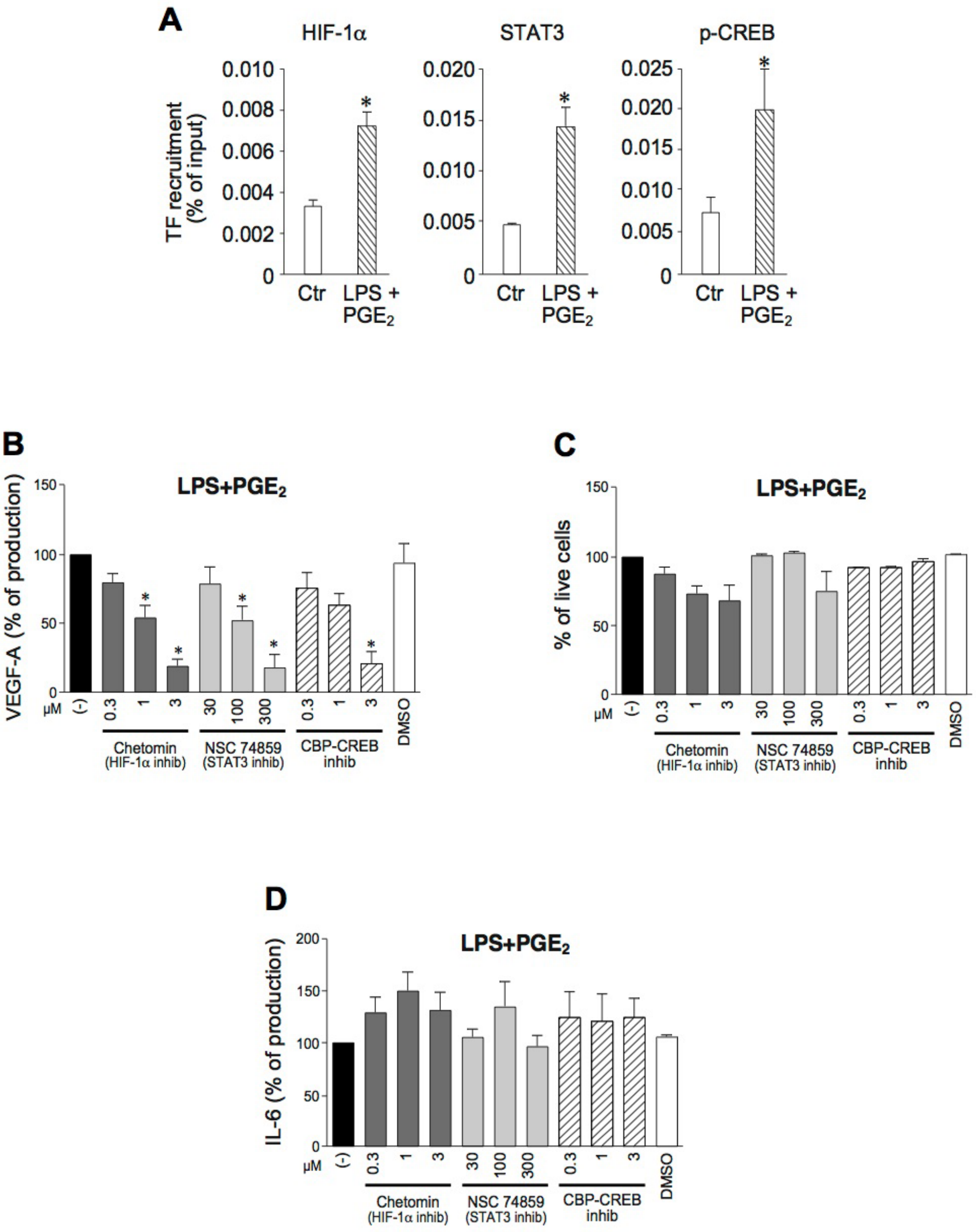

Supplemental Figure 3. The pro-inflammatory secretion of VEGF-A requires the concomitant activation of HIF-1α, STAT3 and CREB.

(A) DCs were stimulated with LPS+PGE_2 for 3 hours. Sonicated nuclear fractions were subjected to chromatin immunoprecipitation with anti-HIF-1α, -phospho-CREB and -STAT3 antibodies and then analyzed by real-time PCR. Data are shown as immunoprecipitated percentage of input DNA and are expressed as mean $\pm$ SEM (n=3); * $P < 0.05$ by Student's *t* test. **(B-D)** DCs were pre-treated for 1 hour with Chetomin, NSC 74859 or CBP-CREB inhibitor and stimulated with LPS+PGE_2 for 24 hours. VEGF-A **(B)** or IL-6 **(D)** production was evaluated by ELISA. **(C)** Cell viability was evaluated by Propidium Iodide staining. Data are expressed as mean $\pm$ SEM (n=3); *$P < 0.05$ by one-way ANOVA with Dunnet's post hoc test.
